# Efficacy and safety of transcutaneous auricular vagus nerve stimulation for frequent premature ventricular complexes: rationale and design of the TASC-V trial

**DOI:** 10.1186/s12906-024-04568-1

**Published:** 2024-07-29

**Authors:** Yu Liu, Xinyao Wei, Lixin Wang, Yanling Yang, Liya Xu, Tianheng Sun, Li Yang, Song Cai, Xiaojie Liu, Zongshi Qin, Lulu Bin, Shaoxin Sun, Yao Lu, Jiaming Cui, Zhishun Liu, Jiani Wu

**Affiliations:** 1 Guang’anmen Hospital, China Academy of Chinese Medical Sciences, Beijing, 100053 China; 2 China Center for Evidence Based Traditional Chinese Medicine, China Academy of Chinese Medical Sciences, Beijing, 100102 China; 3 Department of Psychiatry, Massachusetts General Hospital, Harvard Medical School, Charlestown, MA 02129 USA; 4 Peking University Third Hospital Yanqing Hospital, Beijing, 102199 China; 5 Beijing Longfu Hospital, Beijing, 100010 China; 6 Peking University Clinical Research Institute, Beijing, 100083 China

**Keywords:** Transcutaneous auricular vagus nerve stimulation, Frequent premature ventricular complexes, Randomized controlled trials

## Abstract

**Background:**

Premature Ventricular Complexes (PVCs) are very common in clinical practice, with frequent PVCs (more than 30 beats per hour) or polymorphic PVCs significantly increasing the risk of mortality. Previous studies have shown that vagus nerve stimulation improves ventricular arrhythmias. Stimulation of the auricular distribution of the vagus nerve has proven to be a simple, safe, and effective method to activate the vagus nerve. Transcutaneous au ricular vagus nerve stimulation (taVNS) has shown promise in both clinical and experimental setting for PVCs; however, high-quality clinical studies are lacking, resulting in insufficient evidence of efficacy.

**Methods:**

The study is a prospective, randomized, parallel-controlled trial with a 1:1 ratio between the two groups. Patients will be randomized to either the treatment group (taVNS) or the control group (Sham-taVNS) with a 6-week treatment and a subsequent 12-week follow-up period. The primary outcome is the proportion of patients with a ≥ 50% reduction in the number of PVCs monitored by 24-hour Holter. Secondary outcomes include the proportion of patients with a ≥ 75% reduction in PVCs, as well as the changes in premature ventricular beats, total heartbeats, and supraventricular premature beats recorded by 24-hour Holter. Additional assessments compared score changes in PVCs-related symptoms, as well as the score change of self-rating anxiety scale (SAS), self-rating depression scale (SDS), and 36-item short form health survey (SF-36).

**Discussion:**

The TASC-V trial will help to reveal the efficacy and safety of taVNS for frequent PVCs, offering new clinical evidence for the clinical practice.

**Trial registration:**

Clinicaltrials.gov: NCT04415203 (Registration Date: May 30, 2020).

**Supplementary Information:**

The online version contains supplementary material available at 10.1186/s12906-024-04568-1.

## Background

Premature ventricular complexes (PVCs) are the ectopic beats of the ventricular muscle, which are very common even in population without any abnormal structure of the heart. The detection of PVCs is 40–75% through the 24-hour or 48-hour Holter [1, 2], and that figure for frequent PVCs is around 7.7% through 24-hour Holter [2]. Predictor studies have shown that low education status, smoking, large waist-to-hip ratio, less exercise, taller height, older age, etc. were associated with an increased risk for [3, 4]. Frequent PVCs are stated as no less than 1 PVCs detected by a 12-lead Electrocardiograph, or more than 30 PVCs per hour [5]. More and more studies have revealed that in population with no abnormalities in cardiac structure, frequent PVCs affected the function and structure of left atrial and left ventricular disadvantagely [6], and were associated with the increased risk of cardiovascular risk or death [4, 5, 7]. Patients with frequent PVCs are usually with no symptoms. However, some of them could have desultory fatigue, dizziness, labored breathing and palpitations [8]. More serious, continuously developed PVCs can lead to ischemic cardiomyopathy, non-persistent or persistent ventricular tachycardia, and even heart failure [9]. β-blockers and non-dihydropyridine calcium channel blockers are still the first-line medicines for PVCs [10]. Moreover, the applicable population and side-effects should be noticed. Catheter ablation would be suggested if medication failed. Nonetheless, the application is limited due to its strict indications and high price. Therefore, it is worthwhile to seek a therapy that is effective, well-tolerant, and with few side effects in addition to agents.

Increasing research indicated that there was rich vagal innervation in the ventricle [11], especially in the ventricular myocardium and the ventricular conduction system [12]. It has been shown that vagus nerve stimulation can improve ventricular rhythm [13]. The auricular branch is the only cutaneous mucocutaneous branch of the vagus nerve, and the stimulation in the ear’s distribution area could effectively activate the vagus nerve [14].

Transcutaneous auricular vagus nerve stimulation (taVNS) is a non-invasive and safe physical therapy, which could stabilize the heart rhythm by stimulating the vagus nerve [15]. The vagus nervous system belongs to the autonomic nervous system, and the heart is regulated by the autonomic nervous system. Dysregulation of the autonomic nervous system, such as decreased vagal activity and increased sympathetic activity, can lead to arrhythmias and more serious cardiovascular diseases [16]. The autonomic nervous system can be regulated by taVNS, which could further enhance the excitability of the vagus nerve, inhibit the activity of the sympathetic nerve, and bring the autonomic nervous system to a balanced state [16]. In addition, taVNS has been shown to reduce ventricular interstitial fibrosis, reduce the possibility of ventricular arrhythmias, and stabilize the electrophysiological conduction of cardiomyocytes in models of heart diseases such as myocardial infarction and heart failure [17]. The auricular point CO15 (Xin) is in the concha cavity, and HX1 (Erzhong) is in the middle point of the helix, both are in the area of auricular vagus nerve distribution [18]. Stimulating on these two points can modulates the excitability of the vagus nerve [19]. Besides, laboratory studies have shown that needling in PC6 (Neiguan) and HT7 (Shenmen) stabilizes heart rate by blocking ion channels in tachyarrhythmia cardiomyocytes and stimulating parasympathetic nerves [20–22].

Although there was clinical and experimental basis of taVNS for PVCs, but the high-quality clinical studies are limited with insufficient evidence of efficacy. This study preliminarily evaluated the efficacy of taVNS in reducing the number of PVCs in patients with frequent PVCs, and evaluated the acceptability and safety of patients, laying a foundation for further clinical research.

## Methods

### Study design

**Fig. 1:**
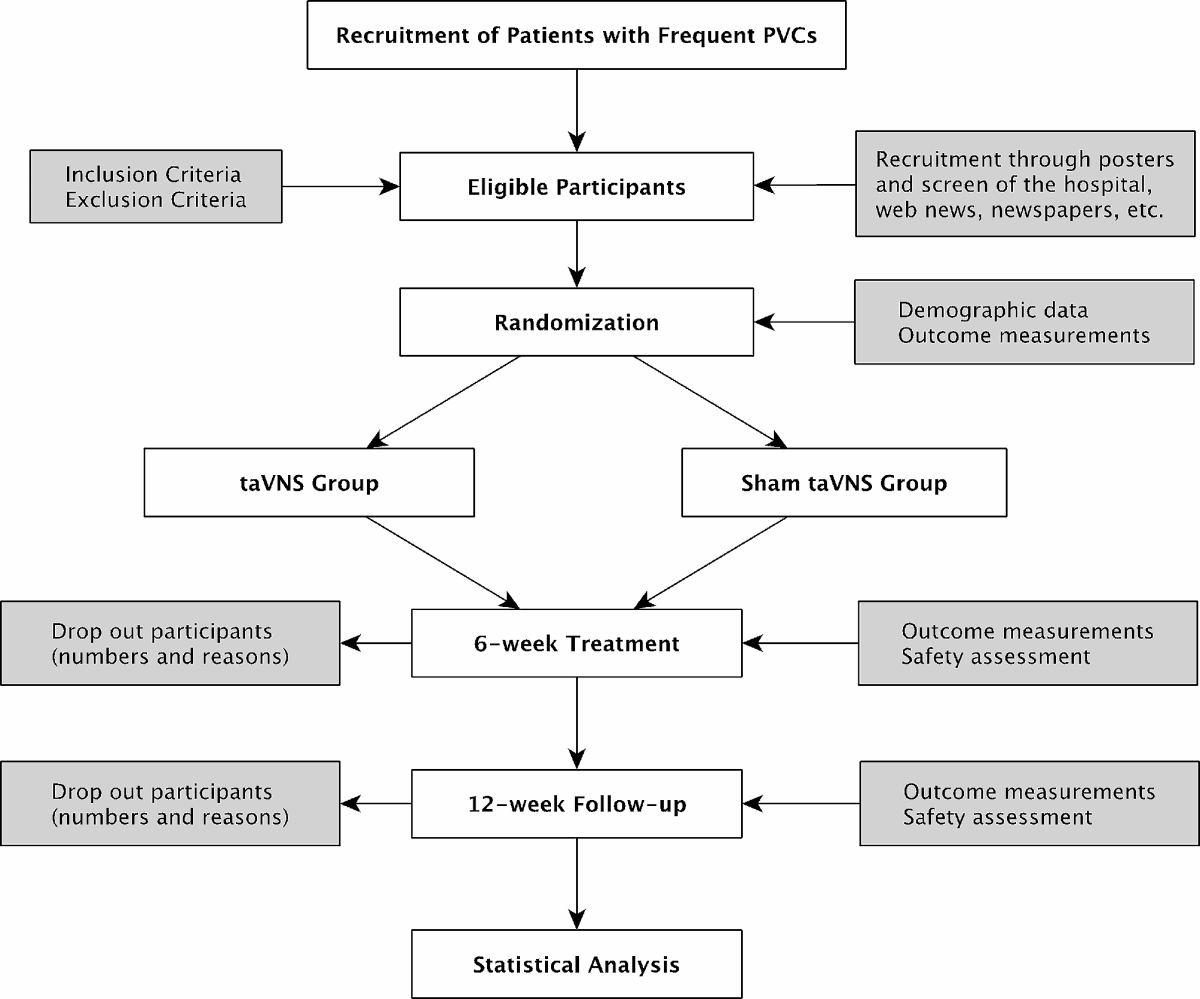
Flowchart of Study PVCs: premature ventricular complexes; taVNS: Transcutaneous auricular vagus nerve stimulation

This is a randomized controlled trial, which is carrying out at three centers in China: Guang’anmen Hospital, China Academy of Chinese Medical Sciences, Peking University Third Hospital Yanqing Hospital and Beijing Longfu Hospital. This trial was started on June 4th, 2020, and will be completed in June 2024 anticipatedly. The flow chart and flow sheet are showed in Fig. 1; Table 1.

**Table 1:** Study schedule by time

Period	Baseline	Treatment						Follow-up		
**Week**	**-1**	**1**	**2**	**3**	**4**	**5**	**6**	**10**	**14**	**18**
Screening	**×**									
Informed consent	**×**									
Demographic data	**×**									
Eligibility	**×**									
Factors that affect heart rate	**×**	**×**	**×**	**×**	**×**	**×**	**×**	**×**	**×**	**×**
History of present illness	**×**									
Previous treatment history	**×**									
Vital signs	**×**	**×**	**×**	**×**	**×**	**×**	**×**	**×**	**×**	**×**
Complete blood analysis	**×**									
Cardiac B ultrasound	**×**									
24-hour Holter	**×**						**×**			**×**
Total cardiac impulse by 24-h Holter	**×**						**×**			**×**
Premature ventricular contractions by 24-h Holter	**×**						**×**			**×**
Supraventricular premature contractions by 24-h Holter	**×**						**×**			**×**
Combined treatment/medication	**×**	**×**	**×**	**×**	**×**	**×**	**×**	**×**	**×**	**×**
VAS for PVC-related symptoms	**×**	**×**	**×**	**×**	**×**	**×**	**×**	**×**	**×**	**×**
Self-rating anxiety scale (SAS)	**×**						**×**			**×**
Self-rating depression scale (SDS)	**×**						**×**			**×**
The short form health survey (SF-36)	**×**						**×**			**×**
Evaluation of treatment associated adverse events		**×**	**×**	**×**	**×**	**×**	**×**			
Evaluation of adherence							**×**			
Evaluation of acceptability							**×**			

### Randomization

The random number was generated by a statistician from Clinical Pharmacological Assessment Center of Guang’anmen Hospital by using SAS software through PROC PLAN statement. The random codes were sealed in opaque envelopes and be placed in order according to the serial numbers outside. A staff who was not involved in the treatment and evaluation of this trial keeps these envelopes and is responsible for the assignment.

### Blinding

Acupuncturists could not be blinded for that the taVNS is a manipulating intervention. The participants, outcome evaluator and statistician are blinded. Patients will accept taVNS in the treatment group, and sham-taVNS without any current output in the controlled group. For the blinding, patients in both groups were told that the feelings of stimulation they would receive might vary individually, with some could feel it and others could not feel it at all. During the treatment, the sound of adjusting the current was the same in both groups.

### Participants

Patients with PVCs will be evaluated for the enrollment. The inclusion criteria contain: (1) diagnosed as the frequent premature ventricular contraction (>720 PVCs through 24 h-Holter) [5] with the Lown grading system of 2, 3 and 4 A [23]; (2) 18 years ≤ age ≤ 75 years; (3) volunteered to participant. Patients will be excluded if they (1) have severe valvular heart disease, congenital heart disease, pericardial disease, hypertrophic cardiomyopathy, unstable angina, acute myocardial infarction, myocarditis, aneurysm, congestive heart failure decompensation period (New York Heart Association classification of III or VI), cardiogenic shock, cerebrovascular disease, hematopoietic system disease, severe mental disease; (2) have bradycardia, including pathologic sinus node syndrome, atrioventricular block no less than the second degree; (3) have already had pacemaker or percutaneous coronary intervention, or who plan to have pacemaker or percutaneous coronary intervention; (4) are female during pregnancy or lactation; (5) have sensory loss; (6) are allergic to electric current or transcutaneous patches; (7) blood pressure ≤ 90/60 mmHg; (8) participate in other clinical trials within 3 months before the screening.

### Intervention

**Fig. 2:**
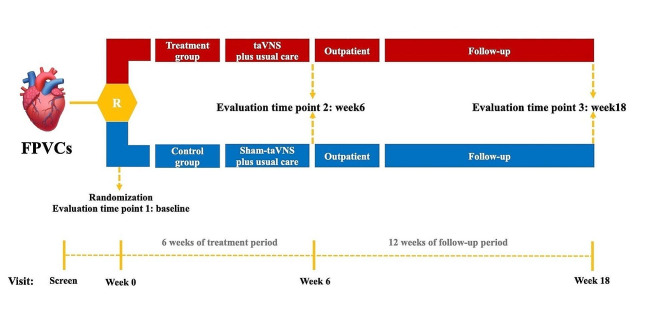
Study periods FPVCs: Frequent premature ventricular complexes; R: Randomization; taVNS: transcutaneous auricular vagus nerve stimulation

**Fig. 3:**
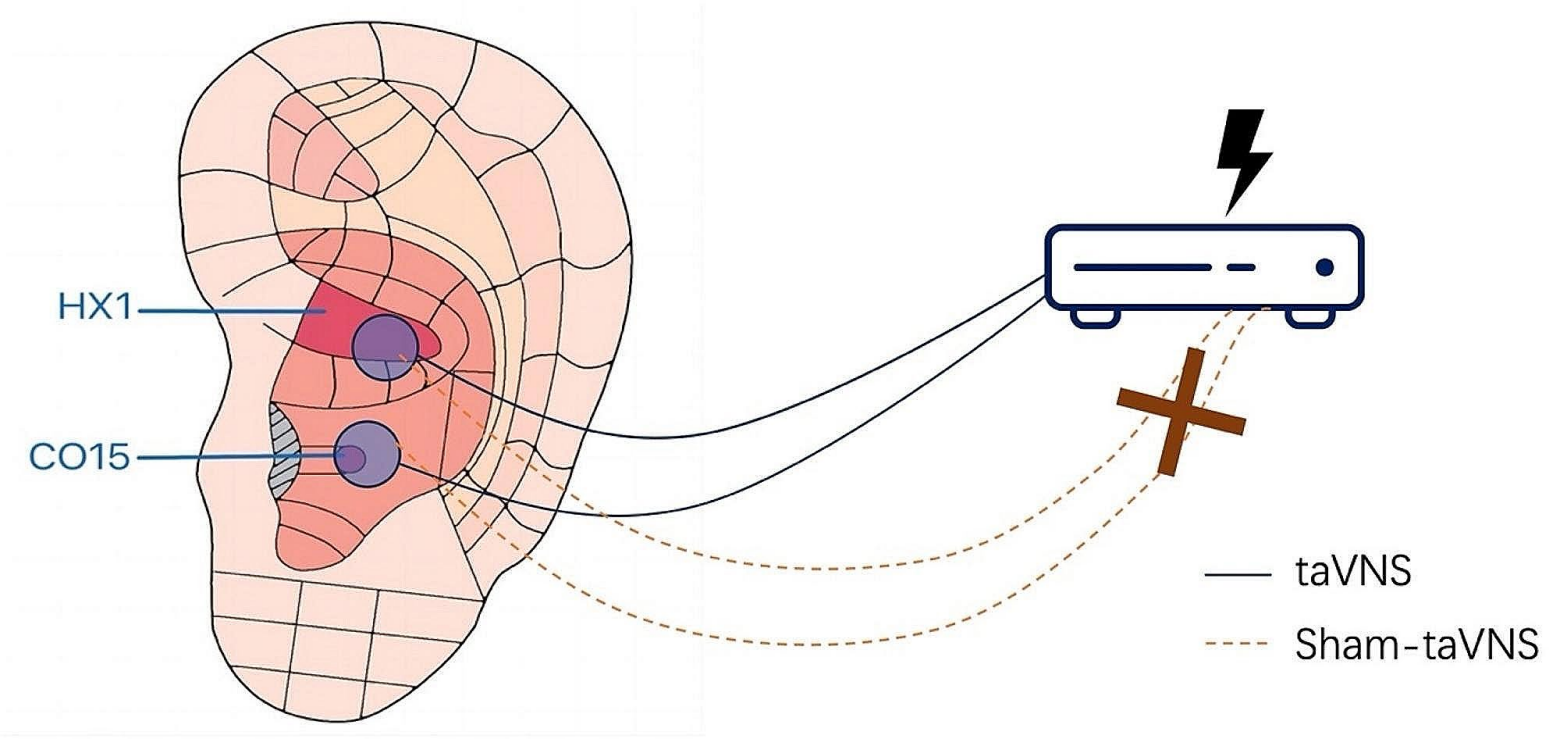
Auricular points stimulation of taVNS and sham-taVNS groups HX1: auricular point of Erzhong; CO15: auricular point of Xin; taVNS: transcutaneous auricular vagus nerve stimulation

Two to three acupuncturists from each site with no less than 2 years of acupuncture experience manipulate the taVNS or sham-taVNS. All acupuncturists are the registered practitioners of traditional Chinese medicine. The acupuncturists and outcome evaluators received a systemic training before the recruitment. Both treatments are constitutive of 30 sessions over 6 weeks with 30 min for each session, 5 sessions per week. Huato disposable ear clip electrodes, skin electrodes and Huato SDZ-IIB stimulator will be used (Suzhou Medical Appliance, Suzhou, China). Intervention procedures of the taVNS and sham-taVNS are showed in Figs. 2 and 3.

#### taVNS group

Participants in taVNS group will receive taVNS and usual care. TaVNS will be performed on HX1(Erzhong) and CO15(Xin). Ear stimulation acupoints are selected according to auricular acupuncture point with the distribution of vagus nerve [18, 24]. Acupoints of PC6 (Neiguan) and HT7 (Shenmen) will also be stimulated to calm the mind. Ear clip electrodes will be attached to bilateral Erzhong and Xin, skin electrodes will be attached to bilateral PC6 and HT7 with a 10 Hz continuous wave, setting the pulse width of 0.2ms ± 30%.

Usual Care: participants will be encouraged to maintain their routine life if they did not take any medicine; otherwise, they will be allowed to continue their usual medicine treatment for PVCs recommended by the guideline. The usual care used by the patients was not allowed to alter from the whole study period.

#### Sham-taVNS group

Participants in Sham-taVNS group will receive Sham-taVNS and usual care. The points, stimulator and stimulating parameters used in Sham-taVNS group are the same with taVNS group. However, we cut the inner electric wire of the stimulator to prevent the current output. Usual Care: the same with the taVNS group.

### Outcome

The primary outcome is the proportion of participants with a ≥ 50% decrease of PVCs from baseline at the end of week 6. The PVCs will be assessed by a 24-h Holter. We will calculate the decrease of PVCs between baseline and week 6 (at the end of the treatment) and obtain the proportion of patients with a decrease of 50%.

Secondary outcomes include: (1) the proportion of participants with a ≥ 50% decrease of PVCs from baseline at the end of week 18 and the proportion of participants with a ≥ 75% decrease of PVCs from baseline at the end of week 6 and 18 which will be assessed the same way as the primary outcome; (2) the change from baseline in PVCs, total cardiac impulse and supraventricular premature contractions at the end of week 6 and 18 which will also be assessed through a 24-h Holter; (3) the score change from baseline in the symptom of palpitation, chest tightness, dizziness or insomnia at the end of week 6 and 18; the change of the proportion of participants with moderate/severe symptoms of palpitation, chest tightness, dizziness or insomnia from baseline at the end of week 6 and 18; The symptom of palpitation will be assessed by a 10-scale VAS from 0 to 10, a higher score indicates a more severe symptom; Score 4–6 are defined as the moderate level, Score 7–10 are defined as the severe level; (4) the score change from baseline in the Self Anxiety Scale (SAS) at the end of week 6 and 18. SAS contains 20 items scoring from 25 to 100. The higher the score significates the greater anxiety. (5) the score changes from baseline in the Self Depression Scale (SDS) at the end of week 6 and 18. SDS contains 20 items scoring from 25 to 100. The higher the score significates the greater depression. (6) the score changes from baseline in 36-Item Short Form Health Survey (SF-36) at the end of week 6 and 18. The SF-36 questionnaire contains eight scales with two measures: physical and mental health. The physical health includes four scales of physical functioning, role-physical, bodily pain, and general health. The mental health is composed of vitality, social functioning, role emotional, and mental health. SF-36 scores from 0 to 100, higher scores indicate better health status.7) subgroup analysis of ages (classified as ≤ 40 years and > 40 years), sex and the severity of PVCs (classified as Grade 2, 3, and 4 A according to Lown grading system) on the primary outcome. 8) the proportion of participants with treatment-related adverse events between two groups.

### Sample size calculation

The null hypothesis is that there would be no difference between the two groups in the proportion of participants with a ≥ 50% reduction of PVCs from baseline after 6 weeks of treatment, and the alternative hypothesis is that there would be a significant difference between the two groups. In our pilot study, the proportion in taVNS and sham-taVNS groups were 58.3% and 21.1%, separately. Ninety participants are required (45 in each group) with a two-tailed test setting power as 90% and alpha as 5%, and considering the rate of loss to follow-up as 15%. To balance the three sites with two groups, 30 participants need to be enrolled in each site (15 per group in one site).

### Statistical analysis

All analyses will be performed using the intention-to-treat population and a *p* value of 0.05 will be taken to indicate a statistically significant effect. The primary analysis will seek to demonstrate the ratio difference of participants with a ≥ 50% decrease of PVCs from baseline at the end of week 6 between taVNS and sham taVNS groups. We will use a generalized linear mixed model to estimate the differences from baseline and between groups. The random effect for participants will be included, the fixed effects include 1 with-subject variable (time point) and 1 between-subject variable (group). Other secondary outcomes with the same pattern of longitudinal data will be analyzed use the similar way as primary analysis. The intervention effect will be summarized as the ratio difference and its 95% confidence interval. For continuous process indicators such as Holter related outcomes and patient reported outcome measures (SAS, SDS, SF-36), we will use mixed effect linear model. A detailed analysis plan including mock tables will be completed before unblinding. For missing data of primary outcome, the multiple imputation method will be used under the missing-at random assumption to generate 100 imputed data sets. All analyses and summaries will be performed by using SAS version 9.4.

### Quality control

Prior to the trial, all staff underwent a special training on the purpose and content of the trial, treatment strategies and quality control. Monitors will check case report forms once every week as well as the acupuncture operation during the treatment period. Dropouts and withdrawals including the reasons will be detailed documented through the trial. Participants’ information will be stored in locked file cabinets at the study sites with limited access; only investigators have the right to access the data. All investigators will always maintain a strict privacy policy to protect confidentiality before, during and after the trial.

## Discussion

In this study, we use taVNS plus usual care, controlled with sham-taVNS plus usual care to initially access the short-term and long-term effect of taVNS for frequent PVCs. Combined with the clinical symptoms of frequent premature ventricular contractions and the reasons that anxiety and depression are closely related to arrhythmia, the PVCs-related symptoms VAS score, SAS score and SDS score are selected as the outcomes in this study aside from the 24-hour Holter, which can improve the report of relevant results. This study would provide clinical practice evidence for the treatment of frequent PVCs, and may help the physicians to make clinical decisions and discover relevant clinical treatments of ventricular arrhythmia. The results of the study will be published after completion of the study.

The regulation of the cardiac function is significantly influenced by the vital role played by the vagus nerve. From the anatomical structure, the postganglionic efferent vagus nerve is distributed throughout most of the cardiac system including the atria, the sinoatrial node, the atrioventricular node, the ventricles, et al. [12]. Stimulation of the vagus nerve can reduce the cardiac rate, atrioventricular conduction and ventricular contractility [25]. Clinical studies have revealed that vagus nerve stimulating can relieve the atrial fibrillation and heart failure by improving the heart rate variability and the left ventricular ejection fraction [26–28], which is also supported by animal research [29–31]. Although not as much as the research on atrial fibrillation and heart failure, there are also some animal studies focusing on the mechanism of vagus nerve stimulation for the ventricular arrhythmia. Vagus nerve stimulation could inhibit the enhancement of sympathetic nerve activity by ventricular muscle [32] and reverse the ventricular arrhythmias induced by high sympathetic nerve stimulation, especially inhibit the ventricular fibrillation and stabilize the ventricular electrophysiological changes [33]. However, this protective effect on the ventricle can also be affected by some drugs, such as atropine and β-blockers [34]. Besides, the vagus nerve was able to regulate the NO release, which could contract blood vessels, by the ventricular myocytes in order to improve the myocardial systolic and diastolic function, increase coronary blood flow, and improve cardiac function [35]. In terms of ventricular electrophysiology, vagus nerve could reduce the maximum slope of the electrocardiogram recovery curve and increase the effective refractory period in the excitatory cycle of ventricular myocytes [36]. Due to the risk and complexity of ventricular arrhythmias, there are relatively few clinical studies. For the vagus nerve stimulation for PVCs, especially the taVNS for PVCs, we only searched out a study protocol, which was registered in 2021, later than our registration.

Recent studies have found that as the only branch of the vagus nerve on the body surface, the afferent nerve fibers of the auricular vagus nerve are consistent with the central projection of the cervical vagus nerve [37], and stimulation of the auricular branch of the vagus nerve can also cause the activation of the vagus nervous system. Therefore, taVNS is a safe and effective treatment with high acceptance by patients. In order to implement blinding as successfully as possible, the proper setup of sham taVNs is crucial. Currently, there are mainly two approaches for setting the sham taVNS. One involves applying identical electrical stimulation as the treatment group in areas with no vagus nerve distribution, such as the auricle or the earlobe [38–42]; another entails placing electrodes in the same locations as the treatment group with no electric current [43], which is also adopted in our study. The key point is that before the treatment, participants will be informed that some of the taVNs might be imperceptible, and they may or may not experience any feeling.

Our study has medical limitations. First, due to the small sample size and short follow-up period in this trial, clinical studies with larger sample size and longer follow-up results will be still needed for the evidence support. Second, in terms of blinding, the operator needs to intervene for the patient, so it is impossible to blind the operator. Third, we did not include a waiting-list group; therefore, the natural course of the disease could not be observed. This study will serve as a preliminary evaluation to lay the foundation for further high-quality clinical research.

## Conclusion

The TASC-V trial will reveal the efficacy and safety of taVNS for frequent PVCs, and provide new clinical evidence for the clinical practice.

### Electronic supplementary material

Below is the link to the electronic supplementary material.

Supplementary Material 1

## Data Availability

No datasets were generated or analysed during the current study.

## Abbreviations

PVCs: premature ventricular complexes
taVNS: transcutaneous auricular vagus nerve stimulation
SAS: self anxiety scale
SDS: self depression scale
SF-36: 36-item short form health survey

## References

[1] Ng GA. Treating patients with ventricular ectopic beats. Heart. 2006;92(11):1707–12. 10.1136/hrt.2005.067843 17041126 10.1136/hrt.2005.067843 PMC1861260

[2] Dong Y, Li X, Zheng W, et al. Prevalence and heart rate variability characteristics of premature ventricular contractions detected by 24-hour Holter among outpatients with palpitations in China: a cross-sectional study. BMJ Open. 2022;12(8):e059337. 10.1136/bmjopen-2021-059337. [published Online First: 20220802]. 35918118 10.1136/bmjopen-2021-059337 PMC9351320

[3] Marcus GM. Evaluation and management of premature ventricular complexes. Circulation. 2020;141(17):1404–18. 10.1161/circulationaha.119.042434. [published Online First: 20200427]. 32339046 10.1161/circulationaha.119.042434

[4] von Rotz M, Aeschbacher S, Bossard M, et al. Risk factors for premature ventricular contractions in young and healthy adults. Heart. 2017;103(9):702–07. 10.1136/heartjnl-2016-309632. [published Online First: 20161021]. 27798051 10.1136/heartjnl-2016-309632

[5] Al-Khatib SM, Stevenson WG, Ackerman MJ, et al. 2017 AHA/ACC/HRS Guideline for management of patients with ventricular arrhythmias and the Prevention of Sudden Cardiac Death: executive summary: a report of the American College of Cardiology/American Heart Association Task Force on Clinical Practice guidelines and the Heart Rhythm Society. Circulation. 2018;138(13):e210–71. 10.1161/cir.0000000000000548 29084733 10.1161/cir.0000000000000548

[6] Alimi H, Bigdelu L, Poorzand H, et al. Assessment of functional and structural echocardiography parameters in patients with frequent premature ventricular contractions without structural heart disease. ARYA Atheroscler. 2022;18(2):1–7. 10.48305/arya.2022.16310 36819835 10.48305/arya.2022.16310 PMC9931611

[7] Priori SG, Wilde AA, Horie M, et al. HRS/EHRA/APHRS expert consensus statement on the diagnosis and management of patients with inherited primary arrhythmia syndromes: document endorsed by HRS, EHRA, and APHRS in May 2013 and by ACCF, AHA, PACES, and AEPC in June 2013. Heart Rhythm. 2013;10(12):1932–63. 10.1016/j.hrthm.2013.05.014. [published Online First: 20130830]. 24011539 10.1016/j.hrthm.2013.05.014

[8] Liu Q, Qin F, Liu N, et al. Is the new risk factor algorithm accurate to predict frequent premature ventricular contraction-induced cardiomyopathy? Int J Cardiol. 2017;247:27. 10.1016/j.ijcard.2017.03.067 28916067 10.1016/j.ijcard.2017.03.067

[9] Lee GK, Klarich KW, Grogan M, et al. Premature ventricular contraction-induced cardiomyopathy: a treatable condition. Circ Arrhythm Electrophysiol. 2012;5(1):229–36. 10.1161/circep.111.963348 22334430 10.1161/circep.111.963348

[10] Al-Khatib SM, Stevenson WG, Ackerman MJ, et al. 2017 AHA/ACC/HRS guideline for management of patients with ventricular arrhythmias and the prevention of sudden cardiac death: a report of the American College of Cardiology/American Heart Association Task Force on Clinical Practice guidelines and the Heart Rhythm Society. Heart Rhythm. 2018;15(10):e73–189. 10.1016/j.hrthm.2017.10.036. [published Online First: 20171030]. 29097319 10.1016/j.hrthm.2017.10.036

[11] Ng GA. Vagal modulation of cardiac ventricular arrhythmia. Exp Physiol. 2014;99(2):295–9. 10.1113/expphysiol.2013.072652. [published Online First: 20130906]. 24014808 10.1113/expphysiol.2013.072652

[12] Capilupi MJ, Kerath SM, Becker LB. Vagus nerve stimulation and the Cardiovascular System. Cold Spring Harb Perspect Med. 2020;10(2). 10.1101/cshperspect.a034173. [published Online First: 20200203]. 10.1101/cshperspect.a034173 PMC6996447 31109966

[13] Weiss T, Lattin GM, Engelman K. Vagally mediated suppression of premature ventricular contractions in man. Am Heart J. 1975;89(6):700–7. 10.1016/0002-8703(75)90184-2 48331 10.1016/0002-8703(75)90184-2

[14] Lai Y. Prevention of noninvasive vagal nerve stimulation against doxorubicin-induced cardiotoxicity and the underlying mechanism. *Wuhan University*. 2020.

[15] Annoni EM, Xie X, Lee SW, et al. Intermittent electrical stimulation of the right cervical vagus nerve in salt-sensitive hypertensive rats: effects on blood pressure, arrhythmias, and ventricular electrophysiology. Physiol Rep. 2015;3(8). 10.14814/phy2.12476 10.14814/phy2.12476 PMC4562562 26265746

[16] Lai Y, Yu L, Jiang H. Autonomic neuromodulation for preventing and treating ventricular arrhythmias. Front Physiol. 2019;10:200. 10.3389/fphys.2019.00200. [published Online First: 20190311]. 30914967 10.3389/fphys.2019.00200 PMC6421499

[17] Jiang Y, Po SS, Amil F, et al. Non-invasive low-level Tragus Stimulation in Cardiovascular diseases. Arrhythm Electrophysiol Rev. 2020;9(1):40–6. 10.15420/aer.2020.01 32637119 10.15420/aer.2020.01 PMC7330730

[18] Auricular Acupuncture Point (WFAS STANDARD-002. 2012). *World Journal of Acupuncture Moxibustion* 2013;23(3)::12–21.

[19] Rogers RC, Hermann GE. Central connections of the hepatic branch of the vagus nerve: a horseradish peroxidase histochemical study. J Auton Nerv Syst. 1983;7(2):165–74. 10.1016/0165-1838(83)90044-9 6875186 10.1016/0165-1838(83)90044-9

[20] Gao J, Zhao Y, Wang Y, et al. Anti-arrhythmic effect of acupuncture pretreatment in the rats subjected to simulative global ischemia and reperfusion–involvement of intracellular Ca2 + and connexin 43. BMC Complement Altern Med. 2015;15:5. 10.1186/s12906-015-0521-y. [published Online First: 20150205]. 25651793 10.1186/s12906-015-0521-y PMC4323136

[21] Huang Y, Lu SF, Hu CJ, et al. Electro-acupuncture at Neiguan pretreatment alters genome-wide gene expressions and protects rat myocardium against ischemia-reperfusion. Molecules. 2014;19(10):16158–78. 10.3390/molecules191016158. [published Online First: 20141009]. 25302705 10.3390/molecules191016158 PMC6271995

[22] Lomuscio A, Belletti S, Battezzati PM, et al. Efficacy of acupuncture in preventing atrial fibrillation recurrences after electrical cardioversion. J Cardiovasc Electrophysiol. 2011;22(3):241–7. 10.1111/j.1540-8167.2010.01878.x. [published Online First: 20100830]. 20807278 10.1111/j.1540-8167.2010.01878.x

[23] Lown B, Wolf M. Approaches to sudden death from coronary heart disease. Circulation. 1971;44(1):130–42. 10.1161/01.cir.44.1.130. [published Online First: 1971/07/01]. 4104697 10.1161/01.cir.44.1.130

[24] He W, Jing XH, Zhu B, et al. The auriculo-vagal afferent pathway and its role in seizure suppression in rats. BMC Neurosci. 2013;14:85. 10.1186/1471-2202-14-85. [published Online First: 20130809]. 23927528 10.1186/1471-2202-14-85 PMC3751281

[25] Liu C, Jiang H, Yu L, et al. Vagal stimulation and arrhythmias. J Atr Fibrillation. 2020;13(1):2398. 10.4022/jafib.2398. [published Online First: 20200630]. 33024499 10.4022/jafib.2398 PMC7533130

[26] Nearing BD, Libbus I, Carlson GM, et al. Chronic vagus nerve stimulation is associated with multi-year improvement in intrinsic heart rate recovery and left ventricular ejection fraction in ANTHEM-HF. Clin Auton Res. 2021;31(3):453–62. 10.1007/s10286-021-00780-y. [published Online First: 20210216]. 33590355 10.1007/s10286-021-00780-y PMC8184538

[27] De Ferrari GM, Crijns HJ, Borggrefe M, et al. Chronic vagus nerve stimulation: a new and promising therapeutic approach for chronic heart failure. Eur Heart J. 2011;32(7):847–55. 10.1093/eurheartj/ehq391. [published Online First: 20101028]. 21030409 10.1093/eurheartj/ehq391

[28] Stavrakis S, Stoner JA, Humphrey MB, et al. TREAT AF (Transcutaneous Electrical Vagus nerve stimulation to suppress Atrial Fibrillation): a Randomized Clinical Trial. JACC Clin Electrophysiol. 2020;6(3):282–91. [published Online First: 20200129]. 32192678 10.1016/j.jacep.2019.11.008 PMC7100921

[29] Zhang Y, Popovic ZB, Bibevski S, et al. Chronic vagus nerve stimulation improves autonomic control and attenuates systemic inflammation and heart failure progression in a canine high-rate pacing model. Circ Heart Fail. 2009;2(6):692–9. 10.1161/circheartfailure.109.873968. [published Online First: 20090922]. 19919995 10.1161/circheartfailure.109.873968

[30] Jiang Z. Research on the relationship between autonomic nervoussystem and atrial fibrillation (AF) and explore the diagnosis or treatment strategies of AF. Shanghai Jiao Tong Univ 2015.

[31] Sabbah HN, Ilsar I, Zaretsky A, et al. Vagus nerve stimulation in experimental heart failure. Heart Fail Rev. 2011;16(2):171–8. 10.1007/s10741-010-9209-z 21128115 10.1007/s10741-010-9209-z PMC3784341

[32] Kent KM, Smith ER, Redwood DR, et al. Electrical stability of acutely ischemic myocardium. Influences of heart rate and vagal stimulation. Circulation. 1973;47(2):291–8. 10.1161/01.cir.47.2.291 4684930 10.1161/01.cir.47.2.291

[33] Huang J, Qian J, Yao W, et al. Vagus nerve stimulation reverses ventricular electrophysiological changes induced by hypersympathetic nerve activity. Exp Physiol. 2015;100(3):239–48. 10.1113/expphysiol.2014.082842. [published Online First: 20141209]. 25720663 10.1113/expphysiol.2014.082842

[34] Yoon MS, Han J, Tse WW, et al. Effects of vagal stimulation, atropine, and propranolol on fibrillation threshold of normal and ischemic ventricles. Am Heart J. 1977;93(1):60–5. 10.1016/s0002-8703(77)80172-5 831412 10.1016/s0002-8703(77)80172-5

[35] Brack KE, Patel VH, Coote JH, et al. Nitric oxide mediates the vagal protective effect on ventricular fibrillation via effects on action potential duration restitution in the rabbit heart. J Physiol. 2007;583(Pt 2):695–704. 10.1113/jphysiol.2007.138461. [published Online First: 20070712]. 17627986 10.1113/jphysiol.2007.138461 PMC2277035

[36] Ng GA, Brack KE, Patel VH, et al. Autonomic modulation of electrical restitution, alternans and ventricular fibrillation initiation in the isolated heart. Cardiovasc Res. 2007;73(4):750–60. 10.1016/j.cardiores.2006.12.001. [published Online First: 20061215]. 17217937 10.1016/j.cardiores.2006.12.001

[37] Zhenya Wang HJ. Nerual-rebalance and ventricular Arrhythmia Induced by myocardial ischemia. *Advances in Cardiovascular diseases* 2019((02):):268 – 72. 10.16806/ j.cnki.issn.1004-3934.2019.02.032.

[38] Dasari TW, Chakraborty P, Mukli P, et al. Noninvasive low-level tragus stimulation attenuates inflammation and oxidative stress in acute heart failure. Clin Auton Res. 2023;33(6):767–75. 10.1007/s10286-023-00997-z. [published Online First: 20231109]. 37943335 10.1007/s10286-023-00997-z

[39] Zhang Y, Huang Y, Li H, et al. Transcutaneous auricular vagus nerve stimulation (taVNS) for migraine: an fMRI study. Reg Anesth Pain Med. 2021;46(2):145–50. 10.1136/rapm-2020-102088. [published Online First: 20201201]. 33262253 10.1136/rapm-2020-102088

[40] Shi X, Hu Y, Zhang B, et al. Ameliorating effects and mechanisms of transcutaneous auricular vagal nerve stimulation on abdominal pain and constipation. JCI Insight. 2021;6(14). 10.1172/jci.insight.150052. [published Online First: 20210722]. 10.1172/jci.insight.150052 PMC8410029 34138761

[41] Wang L, Zhang J, Guo C, et al. The efficacy and safety of transcutaneous auricular vagus nerve stimulation in patients with mild cognitive impairment: a double blinded randomized clinical trial. Brain Stimul. 2022;15(6):1405–14. 10.1016/j.brs.2022.09.003. [published Online First: 20220921]. 36150665 10.1016/j.brs.2022.09.003

[42] Jiao Y. Study on the clinical mechanism of transcutaneous electricalstimulation of auricular points to inhibit hyperarousal in treating insomnia disorder. China Acad Chin Med Sci. 2022. 10.27658/d.cnki.gzzyy.2022.000056 10.27658/d.cnki.gzzyy.2022.000056

[43] Samuel CA, Ebenezer IS. Exploratory study on the efficacy of reflexology for pain threshold and tolerance using an ice-pain experiment and sham TENS control. Complement Ther Clin Pract. 2013;19(2):57–62. 10.1016/j.ctcp.2013.02.005. [published Online First: 20130306]. 23561061 10.1016/j.ctcp.2013.02.005

